# Differentiating transient from persistent diabetic range hyperglycemia in a cohort of people completing tuberculosis treatment in Dhaka, Bangladesh

**DOI:** 10.1371/journal.pone.0260389

**Published:** 2021-11-23

**Authors:** Yosra M. A. Alkabab, Samanta Biswas, Shahriar Ahmed, Kishor Paul, Jyothi Nagajyothi, Sayera Banu, Scott Heysell

**Affiliations:** 1 University of Virginia Medical Center, Division of Infectious diseases and International Health, Department of Medicine, Charlottesville, VA, United States of America; 2 International Centre for Diarrhoeal Disease Research, Bangladesh (icddr,b) Enteric and Respiratory Infections, Infectious Diseases Division, Dhaka, Bangladesh; 3 The Kirby Institute, University of New South Wales, Surveillance Evaluation Research Program, Kensington, NSW, Australia; 4 Center for Discovery and Innovation, Hackensack University Medical Center, Nutley, New Jersey, United States of America; The Ohio State University College of Medicine, UNITED STATES

## Abstract

**Background:**

In recent non-pandemic periods, tuberculosis (TB) has been the leading killer worldwide from a single infectious disease. Patients with DM are three times more likely to develop active TB and poor treatment outcomes. Single glycemic measurements at TB diagnosis may inaccurately diagnose or mischaracterize DM severity. Data are limited regarding glycemic dynamics from TB diagnosis through treatment.

**Methods:**

Prospective study of glycemia dynamics in response to TB treatment measured glycosylated haemoglobin (HbA1c) in patients presenting to TB screening centres in Bangladesh to determine the prevalence and risk factors of hyperglycemia before and at TB treatment completion.

**Results:**

429 adults with active TB disease were enrolled and divided into groups based on history of DM and initial HbA1c range: normoglycemia, prediabetes, and DM. DM was diagnosed in 37%. At treatment completion,14(6%) patients from the normoglycemia and prediabetes groups had HbA1c>6.5%, thus increasing the prevalence of DM to 39%. The number needed to screen to diagnose one new case of DM at TB diagnosis was 5.7 and 16 at treatment completion in the groups without DM. Weight gain>5% at treatment completion significantly increased the risk of hyperglycemia in the groups without DM at TB diagnosis (95% CI 1.23–26.04, p<0.05).

**Conclusion:**

HbA1c testing prior to and at TB treatment completion found a high prevalence of prediabetes and DM, including a proportion found at treatment completion and commonly in people with a higher percentage of weight gain. Further longitudinal research is needed to understand the effects of TB disease and treatment on insulin resistance and DM complications.

## Introduction

In recent non-pandemic periods, tuberculosis (TB) has been the leading killer worldwide from a single infectious disease [1]. With a quarter of the world’s population infected with *Mycobacterium tuberculosis* (MTB), conservative models have forecasted a 20% increase in TB deaths in the five years after COVID-19 has waned compared to pre-pandemic mortality, in large part due to interruptions in TB diagnostic and therapeutic services [2]. Identifying and managing comorbidities is critical to diagnostic services, including diabetes mellitus (DM) [1, 3]. DM is projected to affect up to 700 million individuals by 2045 [4]. Patients with DM-TB have a higher MTB bacillary burden than patients without DM, leading to a delayed bacillary clearance from the sputum, and subsequently, an increased risk of poor treatment outcomes: death, drug resistance, and relapse [4, 5]. Despite consistent reports that people with DM are three times more likely to develop active TB than people without DM, laboratory diagnostics for DM are rarely performed among people with TB in endemic settings [3].

Complicating the definitive diagnosis of DM in people with TB, however, is the observation that the progression from MTB infection to active disease may cause transient/stress-induced hyperglycemia [6, 7] and worsening glucose tolerance that may be improved with TB treatment [8–10]. Such transient hyperglycemia may be driven by inflammation and progressive immune response to disrupted MTB containment, with the release of tumour necrosis factor-alpha (TNF-α) and interferon-gamma (IFN-γ) causing both low insulin production and increased resistance and possible correlates with severity of TB disease [11–13].

Consequently, determination of DM prevalence at only TB diagnosis may overestimate actual DM/TB burden. However, failure to perform follow-up DM testing may miss a smaller population of individuals whose insulin resistance accelerates following TB treatment. Given that follow-up DM testing at TB treatment completion can be costly or logistically challenging, an improved understanding of phenotypes requiring such follow-up testing may be of considerable benefit for settings with high DM-TB co-burden.

In Bangladesh, the DM burden has steadily increased. It now affects 8.1% of the population, converging with a background of TB prevalence of 221/100,000 people and an estimated 361,000 new TB cases per year [1, 4]. Despite World Health Organization (WHO) recommendations and Bangladesh national guidelines [14] for screening for DM among all adults diagnosed with TB using random blood sugar (RBS)/fasting blood sugar (FBS), this practice is not routinely performed.

Hence, we aimed to prospectively study glycemic dynamics in response to TB treatment as measured by POC HbA1c among a representative population from urban Dhaka enrolled from public-private partnered centres that allowed linkage to DM-TB co-management to: 1- determine the prevalence of prediabetes and DM measured at TB diagnosis compared to the prevalence measured at TB treatment completion and, 2- quantify risk factors for DM development at treatment completion among those without DM at TB diagnosis.

## Methods and materials

### Study setting

International Centre for Diarrhoeal Disease Research, Bangladesh (icddr,b), with the patronage of the National TB Control Program, Bangladesh, has been operating several TB Screening and Treatment Centres (TBSTCs) that were established under the Social Enterprise Model around Dhaka metropolitan city to serve a large and representative proportion of people presenting with TB symptoms [15]. In these TBSTCs located at neighbourhoods of urban Dhaka: Mohakhali, Dhanmondi, Golapbagh, Mirpur, Rampura, Uttara, and Old Dhaka, patients are referred by private and public health providers, pharmacies, word-of-mouth for TB screening, and patients seeking confirmatory TB diagnosis or anti-TB medications if diagnosed elsewhere.

### Clinical procedures

From October 2018 to July 2019, TB patients aged >15 years, men and non-pregnant women with active rifampin-susceptible TB disease, were consecutively approached for enrollment. After taking informed written consent, patients were offered free DM screening using POC HbA1c and RBS/FBS before initiation of anti-TB treatment. Anti TB treatment included daily weight-based dosing of four medications for the induction phase (isoniazid, rifampin, ethambutol and pyrazinamide for two months) followed by two medications (isoniazid and rifampin for four months) for the continuation phase. We collected clinical data, including history of DM. Patients with a history of DM (regardless of their HbA1c status) and patients with no history of DM but with diabetic range hyperglycemia (HbA1c ≥6.5%) were referred for concurrent DM management. All patients enrolled were treated for rifampin-susceptible TB and followed with acid-fast bacillus (AFB) smear microscopy after two and five months and on completion of treatment as per national TB guidelines. Additionally, a repeat HbA1c test was performed at treatment completion.

### Laboratory procedures

POC HbA1c test requires 5 ml of venous blood collected after taking all necessary aseptic precautions by a trained medical technologist. Two varieties of HbA1c analyzer, the Boditech icroma and the Hemocue 501 analyzers, were used and were calibrated at the beginning of the study thereafter yearly.

### Data variables

Data were collected using a tablet-based application during screening and follow-up of participants. Along with recording the HbA1c% against the unique study identifier at the initiation and completion of TB treatment, we collected the socio-demographic and clinical data, such as treatment start and completion date, age, sex, height in centimetres(cm), weight in kilograms(kg), location of TB disease (pulmonary or extra-pulmonary), mode of TB diagnosis (bacteriological or clinical), presence of cough, fever, night sweats, fatigue, loss of appetite, weight loss, previous history of TB, smoking history, and chest X-ray report for evidence of cavities.

### Statistical analysis

Appropriate statistical methods analyzed De-identified data using the IBM Statistical Package for the Social Sciences (SPSS) 27 system. Patients were divided into three groups based on the history of DM and HbA1c levels at enrollment; normoglycemia group (no history of DM and HbA1c <5.7%), prediabetes group (no history of DM and intermediate hyperglycemia; HbA1c <5.7–6.4%), and DM group (history of DM regardless of HbA1c levels and patients with no history of DM however they have HbA1c ≥6.5%). Demographic and baseline clinical characteristics were compared among all patients in the DM and prediabetes groups in relation to those with normoglycemia group (reference group) by using the Chi-Square test or Fisher exact test. Independent t-test or Mann Whitney U test were used for continuous data such as age, weight, body mass index (BMI) and, POC HbA1c% levels. Next, a paired-sample t-test was conducted to compare the change in HbA1c% at enrollment and TB treatment completion. The number needed to screen (NNS) to diagnose one case of new DM in patients with TB disease was also calculated at enrollment and treatment completion.

Binary logistic regression test was used to measure the odds ratio (OR) with 95% confidence intervals (CI) for predictors of the development of diabetic range hyperglycemia (HbA1c ≥ 6.5%) at treatment completion in the normoglycemia and prediabetes groups. Per routine practice, patients with prediabetes at TB treatment initiation were not treated with anti-diabetes pharmacotherapy but managed with diet, lifestyle modification, and close follow-up. Variables that have been previously associated with higher MTB bacillary burden at treatment initiation or more prolonged recovery from TB disease such as, the presence of cavitary lung disease or positive sputum smear microscopy, were included in the regression model, as well as factors that may influence DM disease progression: age, sex and, weight gain of >5% of body weight during TB treatment. All tests of significance were two-tailed (p<0.01).

### Ethical approval

The study protocol, consent forms were reviewed and approved by the institutional review board at icddr,b. Informed written consent was obtained from all participants aged ≥18 years, and assent was taken from participants aged 15–17 years, along with consent from their parents/guardians.

## Results

### Diabetes disease classification at TB diagnosis and treatment initiation:

Total 429 patients with active TB disease presented to the TBSTCs. Based on POC HbA1c% range and history of DM: 120 (28%) were categorized as normoglycemia, 151 (35%) had prediabetes, and 158 (37%) had DM, of which 100 (23% of the total population) had a history of DM and 58 (14% of the total population) had no history of DM, but their HbA1c at treatment initiation was >6.5% (Table 1).

**Table 1:** Patients’ demographics at TB diagnosis.

Characteristic	Normoglycemia* *N =* 120	Prediabetes *N = 151*	*P*-value	DM *N = 158*	*P*-value
Mean age, years	30 ± 12	29 ±13	0.98	45 ± 15	<0.01
15–30	79(66%)	97(64%)	0.91	29(19%)	<0.01
31–45	32(27%)	38(25%)	0.90	48(30%)	0.90
46–60	4(3%)	10(7%)	0.90	57(36%	<0.01
>61	5(4%)	6(4%)	0.99	24(15%)	<0.01
Male, *n* (%)	69 (58%)	92 (61%)	0.83	114 (72%)	0.03
Mean BMI, kg/m^2^	20 ± 4	20 ± 4	0.83	22 ± 4	0.01
Mean weight, kg	52 ± 12	51 ± 12	0.63	57 ± 11	0.01
Haemoptysis, *n* (%)	18 (15%)	29(19%)	0.65	32 (20%)	0.50
Fever, *n* (%)	98(82%)	131 (87%)	0.50	130(82%)	0.99
Weight loss, *n* (%)	102(85%)	114(76%)	0.10	139(88%)	0.80
Smoking, *n* (%)	45(38%)	47(31%)	0.52	54(34%)	0.83
History of DM, *n* (%)	0 (0%)	0 (0%)		100 (63%)	
Diet only	-	-		3	
Meds				97	
Insulin				45	
Metformin				41	
No history of DM + DM range hyperglycemia	-	-		58	
TB location					
EPTB, *n* (%)	36(30%)	33(22%)	0.25	27(17%)	0.03
PTB, *n* (%)	84(70%)	118(78%)	0.25	131(83%)	0.03
Cavity, *n* (%)	16 (13%)	28 (19%)	0.51	32(20%)	0.29
Household TB contacts, *n* (%)	5 (4%)	9 (6%)	0.78	7 (5%)	1.00
Sputum culture, Positive, *n* (%)	80(67%)	104(69%)	0.92	120 (76%)	0.211
GeneXpert Positive, *n* (%)	67 (89%)	98 (93%)	0.74	111 (92%)	0.83
MTB burden, *n* (%)					
Low	27(40%)	37(38%)	0.94	42(38%)	0.94
High	40(60%)	61(62%)	0.94	69(62%)	0.94
Mean RBS, *mmol*	5.5± 1	5.7 ±1.3	0.95	11.1 ±6.0	<0.01
Mean HbA1c%	5.0 ±0.5	6.0 ±0.2	<0.01	9.1± 2.5	<0.01

*Reference for *P-*values.

Normoglycemia = HbA1c <5.9% with no history of diabetes mellitus. Prediabetes = with no history of diabetes and HbA1c 6.0–6.4%. DM: history of diabetes mellitus or HbA1c ≥6.5% regardless of DM history. BMI: Body mass index. TB: tuberculosis. Extrapulmonary TB(EPTB), Pulmonary TB (PTB) MTB: *Mycobacterium tuberculosis*. RBS/FBS: random blood sugar/fasting blood sugar.

Demographics at treatment initiation did not significantly differ between the prediabetes and normoglycemia groups, except for the expectedly higher mean HbA1c% of 6.0±0.2 vs. 5.0±0.5 (p<0.01 95% CI 0.5–1.4). When comparing the demographics of the DM group to the normoglycemia group, however, patients in the DM group were significantly more likely to be male (72% vs 58%, p<0.05, 95% CI 0.01–0.28) and were older with a mean age of 45±15 years in the DM group vs 30±12 years in the normoglycemia group (p<0.01, 95% CI 12–19). Patients in the DM group had a higher mean weight (kg) and BMI (kg/m^2^) compared to the normoglycemia group, with a mean weight of 57±11 vs 52±12 (p<0.01, 95 CI 1.1–7.8) and BMI of 22±4 vs 20±4 (p<0.01, 95 CI 0.3–2.7). The DM group was also significantly more likely to present with pulmonary TB compared to those with normoglycemia. However, sputum culture positivity and the proportion with sputum Xpert MTB/RIF of high MTB DNA burden did not differ significantly among groups. Patients with known DM prior to treatment initiation were managed by lifestyle modifications only in three patients (3%), while the remaining 97 (97%) patients were prescribed anti-diabetes medications, including 45 (45%) patients receiving insulin with or without other oral hypoglycemic agents, and 41 (41%) patients prescribed a metformin-based oral therapy.

### Diabetes disease classification at TB treatment completion

**Fig 1:**
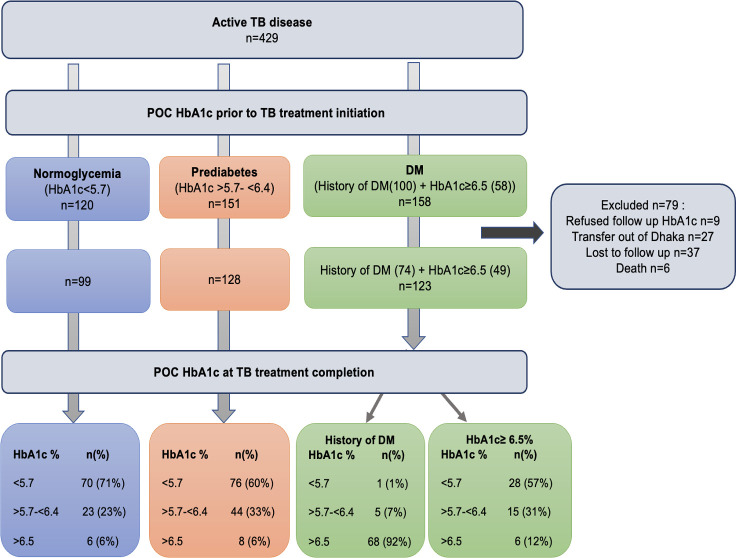
Flow diagram describing patient point-of-care Hemoglobin A1c (POC HbA1c) levels and history of diabetes mellitus (DM) at initiation and completion of tuberculosis (TB) treatment.

Ultimately, 350 (82%) patients had documented POC HbA1c levels at treatment completion (Fig 1). For patients without HbA1c at treatment completion, nine patients refused HbA1c testing, 27 patients were transferred out of Dhaka to another city, 37 patients were lost to follow up, and six patients died (four patients died from the normoglycemia group, and two died from the DM group) prior to treatment completion.

Using the classifications of normoglycemia, prediabetes, and DM from treatment initiation and the normoglycemic group as the reference, the mean HbA1c% for the DM group decreased from 9.1±2.5 at treatment initiation to 8.2±2.7% at treatment completion with a per cent change of -0.15±0.26 (p<0.01 95% CI 0.15–0.30), the prediabetes group decreased from 6.0±0.2 to 5.5±0.6 with a per cent change of -0.09±0.12 (p<0.01 95 CI 0.1–0.2) (Table 2).

**Table 2:** Patient characteristics at tuberculosis (TB) treatment completion grouped by category at TB diagnosis.

Characteristic	Normoglycemia*N = 99	Prediabetes *N = 128*	*P*-value	DM *N = 123*	*P*-value
Mean weight, kg	54 ±12	53 ± 11	0.94	59 ± 12	< 0.01
Mean BMI, kg/m^2^	21±4	21±4	0.94	23±4	< 0.01
Weight gain>5%,*n* (%)	51 (52%)	67 (53%)	1.00	58 (47%)	0.80
Mean weight% change	0.05±0.08	0.06±0.08	0.41	0.05±0.06	0.99
Mean HbA1c% at treatment completion	5.4 ±0.6	5.5 ±0.6	0.92	8.2 ±2.7	<0.01
HbA1c change, *n* (%)					
Decreased	38 (38%)	101 (79%)	<0.01	89 (72%)	<0.01
Increased	57 (58%)	20 (16%)	<0.01	32 (26%)	<0.01
No change	4 (4%)	7 (5%)	0.84	2 (2%)	0.61
Mean HbA1c% change	0.06±0.15	−0.09±0.12	<0.01	−0.15±0.26	<0.01

*Reference for *P-*values.

Normoglycemia = HbA1c <5.9% with no history of diabetes mellitus. Prediabetes = with no history of diabetes and HbA1c 6.0–6.4%. DM: history of diabetes mellitus or HbA1c ≥6.5% regardless of DM history. BMI: Body mass index. TB: tuberculosis.

During TB treatment, patients with diabetic range hyperglycemia with no prior history of DM at treatment initiation were linked to DM care and were managed with lifestyle modifications only. Forty-nine of those patients had follow up POC HbA1c levels; 28 (57%) had normalized glycemia levels (HbA1c<5.7%), 15 (31%) had prediabetes range hyperglycemia (HbA1c>5.7-<6.4%) and only 6 (12%) had persistent diabetes (HbA1c>6.5%). In contrast, six (6%) patients from the normoglycemia group at TB treatment initiation developed diabetic range hyperglycemia (HbA1c >6.5%), and 23 (23%) developed prediabetes range hyperglycemia (HbA1c 5.7–6.4%) at treatment completion. At treatment initiation, a further 8 (6%) patients from the prediabetes group developed diabetic range hyperglycemia at treatment completion (Fig 1). The NNS to diagnose one new case of DM among those with TB at treatment initiation was 5.7 (329/58) compared to the NNS to diagnose one new case of DM at treatment completion of 16 (277/14).

### TB treatment outcomes and predictors of persistent DM range hyperglycemia

Binary logistic regression analysis adjusted for age, sex, presence of cavity on chest x-ray, weight gain of >5%, and positive smear microscopy at two months during TB treatment demonstrated that among patients with normoglycemia and prediabetes at TB treatment initiation, those that gained >5% of their weight at the end of therapy were significantly more likely to develop DM range hyperglycemia at TB treatment completion (adjusted OR 5.51, 95% CI 1.20–24.38, p<0.05). (Table 3)

**Table 3:** Binary logistic regression model for predictors of the development of diabetic range hyperglycemia (HbA1c ≥ 6.5%) at treatment completion in the normoglycemia and prediabetes groups.

Covariate	B	SE.	OR	95% CI	P-value
Age					
<35 years			Reference	Reference	
>35 years	-1.08	1.07	0.34	0.04–2.75	0.31
Cavity on chest imaging					
Negative			Reference	Reference	
Positive	-0.22	0.82	0.81	0.16–4.01	0.79
Sex					
Female			Reference	Reference	
Male	0.50	0.58	9.56	1.65–0.53	0.40
Weight gain at treatment completion					
<5%			Reference	Reference	
>5%	1.71	0.78	5.51	1.20–24.38	0.03
Sputum smear at two months					
Negative			Reference	Reference	
Positive	-0.11	0.23	0.90	0.57–1.39	0.62

SE: Standard error, OR: Odds ratio, CI: confidence interval.

Treatment outcomes were generally favourable. Aside from the six deaths previously mentioned which were not able to have follow-up HbA1c, a total of 31 patients had positive sputum smear microscopy at two months; 13 (8%) patients were from the DM group, nine (6%) from the prediabetes group and, nine (8%) from the normoglycemia group. All patients initially categorized as DM that were not lost to follow-up were linked to DM-TB co-management.

## Discussion

In this prospective observational cohort, we found that more than a third of patients presenting with active TB disease in Dhaka, Bangladesh had DM by utilizing POC HbA1c testing at TB diagnosis. Notably, 14% had diabetic range hyperglycemia despite normal RBS readings. If including patients that developed DM range hyperglycemia at TB treatment completion, a remarkable 39% of the total population had a prior history of DM or DM range hyperglycemia at some point during the treatment interval. Yet, a considerable proportion of hyperglycemia was transient and normalized with TB therapy alone and without other hypoglycemic medication. Hence, the NNS to diagnose one new case of DM at TB treatment initiation was 5.7 but increased to 16 if performing POC HbA1c only at treatment completion. Notably, among those with normoglycemia or prediabetes at treatment initiation, weight gain > 5% during TB treatment significantly increased the risk of developing diabetic range hyperglycemia at treatment completion and maybe a target population for repeat screening.

Our findings demonstrate the limitations of using RBS to screen DM among people with active TB disease and undernutrition and the potential misclassification of DM at TB diagnosis even when using HbA1c levels. Our cohort in Bangladesh may have differed from others performing DM testing at TB diagnosis, which have found a varying prevalence of undiagnosed DM in relative proportion to community DM prevalence [16–20]. Instead, this cohort from urban Dhaka may be more representative of the younger, “lean” phenotype with active TB and hyperglycemia that has been described from the Indian subcontinent [21]. Although patients with TB-DM are usually > 35 years compared to TB patients without DM in other cohorts and in considering classification at treatment initiation in this cohort [4, 16, 18], patients with persistent hyperglycemia at treatment completion were younger with a mean age of <35 years. Besides age, higher BMI (≥25 kg/m^2^) has been identified as a risk factor for DM [22]; however, this threshold may underestimate risk in patients with specific dietary habits, and certainly among wasting diseases such as active TB, where the mean BMI for the DM group in our cohort was only 22±4 [21, 23, 24]. Other markers of body fat distribution, such as waist to hip ratios or mean upper arm circumference, while not measured in this cohort, maybe more correlative with DM disease risk than weight or BMI.

In addition to anthropometrics, insulin levels may help classify the DM disease spectrum more accurately. It has been shown that chronic hyperglycemia impairs the initiation of adaptive immunity and increases susceptibility to TB infection in a murine model of diabetes induced by streptozotocin [25]. The therapeutic effect of anti-TB drugs depends on both adaptive and innate immunity. A drastically upregulated immune system during anti-TB treatment could substantially increase the levels of TNF- α, IFN-γ, and interleukin-17, which could lead to insulin resistance and DM [26]. These changes could be transient, and follow-up studies are needed to examine the link between inflammatory cytokines, hyperglycemia, and anti-TB drug efficiency. More importantly, along with HbA1c levels, fasting glucose and insulin levels may be monitored during treatment. In this cohort, we identified a further 35% of all TB patients with prediabetes at treatment initiation but were otherwise clinically similar to the normoglycemia group. Prediabetes range hyperglycemia was also transient and normalized for the majority at treatment completion, similar to the few other TB cohorts where blood sugar has been prospectively measured [8, 9, 27]. However, long-term studies would be needed to determine if DM or prediabetes range hyperglycemia at TB treatment initiation that normalizes with TB treatment is associated with a higher likelihood of DM disease and DM complications later in life.

Overall, HbA1c levels decreased significantly in more than 70% of patients in the DM and prediabetes TB group, consistent with other studies showing successful reductions of HbA1c levels at TB treatment completion [10, 20, 28]. Weight change had been reported in prior studies to be one of the predictors of treatment response in TB infected patients [29, 30]. In our cohort, weight gain of >5% of body weight was also significantly associated with the development in diabetic range HbA1c in patients not known to have DM. Prior case reports have argued for medication-induced hyperglycemia and insulin resistance from the extensive hepatic metabolization of rifampicin, isoniazid, and pyrazinamide [13, 31–34], which usually disappears after two weeks of stopping TB treatment [13]. Further research measuring glycemia and insulin dynamics beyond TB treatment is ultimately needed to determine if the post-TB DM phenotype is more akin to gestational diabetes [34] or if those patients carry a different risk of developing relapse of TB disease [35, 36].

The strengths of our study include the prospective design, relatively large sample size from a representative population in a DM/TB co-endemic setting, availability and feasibility of POC HbA1c at all TB screening centres, and active patient linkage to DM care and follow-up. However, there were several limitations. We could not confirm the POC HbA1c simultaneously with serum HbA1c levels considering that POC HbA1c results may be influenced by the presence of hemoglobinopathies, history of recent blood loss, or transfusions [16, 37]. Yet, these conditions, as well as underlying chronic medical conditions (such as cardiovascular diseases, asthma or chronic kidney diseases) were not reported and are otherwise less common in this setting and age group. A recent study in Ontario showed a strong positive correlation between serum HbA1c and POC HbA1c [38]; however, in some patients with severe anaemia, POC testing may overestimate HbA1c values by 1.1% [37]. Although patients’ outcomes were mainly favourable, we did not collect more precise time measurements to sputum culture conversion to negative (microbiological cure) as one could with regular sputa collection. Instead, we relied on the programmatic routine, which uses the less frequent smear microscopy during follow-up, a much less sensitive technique for detecting viable bacilli. A larger study with a more rigorous sputum culture may better determine differences between the persistent and transient hyperglycemia groups and their microbiological outcomes.

In conclusion, POC HbA1c detected a considerably high proportion of DM range hyperglycemia among patients presenting with active TB in Dhaka despite relatively young age and lower BMI than other DM-TB cohorts. However, limiting testing for DM to only TB diagnosis potentially misclassified the majority of DM range hyperglycemia, which was instead transient and normalized with TB treatment completion. We suggest a re-testing strategy at treatment completion for patients with DM range hyperglycemia identified at TB diagnosis and those with normoglycemia and prediabetes that gain >5% of weight during treatment. Further longitudinal research is needed to characterize biomarkers that differentiate the transient from the persistent hyperglycemic phenotypes and their correlation with longer-term DM disease and late manifestations of TB infection, including relapse.
